# Variation in the *IL1B*, *TNF* and *IL6* genes and individual susceptibility to prosthetic joint infection

**DOI:** 10.1186/1471-2172-13-25

**Published:** 2012-05-08

**Authors:** Anna Stahelova, Frantisek Mrazek, Matej Smizansky, Martin Petrek, Jiri Gallo

**Affiliations:** 1 Laboratory of Immunogenomics and Immunoproteomics, Faculty of Medicine and Dentistry, Palacky University Olomouc, I. P. Pavlova 6, Olomouc, 77520, Czech Republic; 2 Department of Orthopaedics, Faculty of Medicine and Dentistry, Palacky University Olomouc and University Hospital, I. P. Pavlova 6, Olomouc, 77520, Czech Republic

## Abstract

**Background:**

Prosthetic joint infection (PJI) is an important failure mechanism of total joint arthroplasty (TJA). Here we examine whether the particular genetic variants can lead to increased susceptibility to PJI development.

**Results:**

We conducted a genetic-association study to determine whether PJI could be associated with functional cytokine gene polymorphisms (CGP) influencing on innate immunity response. A case–control design was utilized and previously published criteria for PJI were included to distinguish between cases and control subjects with/without TJA. Six single nucleotide polymorphisms (SNPs) located in the genes for interleukin-1beta (SNP: *IL1B*-511, +3962), tumour necrosis factor alpha (*TNF*-308, -238) and interleukin-6 (*IL6*-174, nt565) were genotyped in 303 Caucasian (Czech) patients with TJA (89 with PJI / 214 without PJI), and 168 unrelated healthy Czech individuals without TJA. The results showed that carriers of the less common *IL1B*−511*T allele were overrepresented in the group of TJA patients with PJI (69%) in comparison with those that did not develop PJI (51%, p = 0.006, p_corr_ = 0.037) and with healthy controls (55%, p = 0.04, p_corr_ = N.S.). There was no significant difference in the distribution of the remaining five investigated CGPs and their haplotypes between groups.

**Conclusion:**

A functional variant of the gene encoding for IL-1beta was preliminarily nominated as a genetic factor contributing to the susceptibility to PJI. Our results should be independently replicated; studies on the functional relevance of *IL1B* gene variants in PJI are also needed.

## Background

Prosthetic joint infection (PJI) is a feared complication of total joint arthroplasty (TJA) regardless of the site. Current estimates suggest that despite careful management to avoid all possible sources of infection, PJI can exhibit in up to 1.7% of primary hip arthroplasties and 2.5% of primary knee arthroplasties [1,2]. Majority of PJI is tightly linked to intraoperative contamination of implanted device. It was repeatedly demonstrated that any medical device implanted into the host body impairs local innate host response facilitating development of PJI [3]. Therefore rigorous preventative measures have been introduced into the clinical practice to diminish intraoperative microbial exposure and increase the likelihood that the host immune response together with antibiotics will tackle remaining bacterial load [4]. In this line, each patient undergoing TJA should have the same chance for PJI. However, majority of patients did not experience PJI; hence there is a question why some patients cannot escape the fate of PJI.

It has been suggested that patients with PJI could have a defect in their immune response [5]. Recognition of the bacteria by pattern-recognition receptors (PRRs) leads to intracellular signal transduction by adaptor molecules that across several cascades induce increased expression of proinflammatory cytokines and their release from inflammasomes [6,7]. Of these the most prominent roles are played by interleukin (IL)-1beta and IL-18 that trigger the complex inflammatory response together with the participation of other proinflammatory mediators such as IL-6 and tumour necrosis factor (TNF) alpha [8]. These cytokines regulate the recruitment, activation, proliferation and differentiation of immune cells in order to eliminate the local microbial burden. Interindividual variability in cytokine production has been observed, and these variations are suspected to be genetically determined, most frequently via the effects of polymorphisms within regulatory regions of the cytokine genes [9-11]. Accordingly, relevant functional polymorphisms in the cytokine genes may be implicated in the pathogenesis of PJI for instance via impairing the effector (post-recognition) phase of the innate immune response. In this study, we hypothesized that frequency of particular variants of the genes encoding for a set of proinflammatory cytokines differs significantly between the patients exhibiting and avoiding PJI.

## Methods

### Study population

Between February 2004 and October 2010, blood samples were collected by venopuncture from 303 Czech patients with TJA. A target group consisted of 89 patients with PJI. Of them majority were the patients that were treated for PJI at the Department of Orthopaedics, University Hospital Olomouc between January 1998 and June 2010 (N = 73). To increase the number of PJIs and thus the power of the study, the orthopaedic departments in Frydek Mistek (N = 11) and Znojmo (N = 5) both in the Czech Republic, were asked to recruit patients treated for PJI at these departments. A control group with TJA (N = 214) was created from patients who did not experience PJI up to the date of blood sampling; they were reoperated at the Department of Orthopaedics, University Hospital Olomouc for aseptic loosening and periprosthetic osteolysis or still had a functional primary prosthesis (Table 1). Majority of patients with total hip arthroplasty (> 80%) had a cementless implant while all knees were cemented.

**Table 1:** Basic characteristics of the patients with total joint arthroplasty (TJA) with prosthetic joint infection (PJI) and those without PJI (aseptic TJA)

	**PJI**	**Aseptic TJA**	**p Value**
**Patients**; N	89	214	
**Gender**; Men/Women	44/45	67/147	p = 0.003
**Age at index surgery**; years	63 (52–69)	47 (42–51)	p < 0.001
**Joint**; hip/knee/other*	39/47/3	202/12/0	p < 10^-5^
**BMI**	29 (26–30)	28 (25–30)	p = 0.3
**Type of PJI:**			
Early	22	n.a.	
Delayed	39	n.a.	
Haematogenous	21	n.a.	
Other	7	n.a.	
**Intraoperative culture:**			
Staphylococci	54	n.a.	
Other pathogens	17	n.a.	
Negative	18	n.a.	
**Primary diagnosis:**			p < 10^-5^
Osteoarthritis	68	58	
Dysplastic joint	7	96	
Inflammatory arthritis	3	10	
Other diagnoses	11	50	
**Comorbidity:**			
DM	11	18	p = 0.29
RA	2	11	p = 0.42
other	21	49	p = 1.0
no	55	136	p = 0.77

Legend: Continuous data presented as median with interquartile (1^st^ to 3^rd^) range in parentheses.

p values for comparison between the groups of patients with/without PJI were calculated by Chi-square test (Pearson’s) with appropriate degrees of freedom (d.f.), Fisher exact test or Mann–Whitney U test according to the type of data. *Shoulder, elbow.

BMI, body mass index; DM, diabetes mellitus; n.a., not applicable; RA, rheumatoid arthritis.

**Figure 1:**
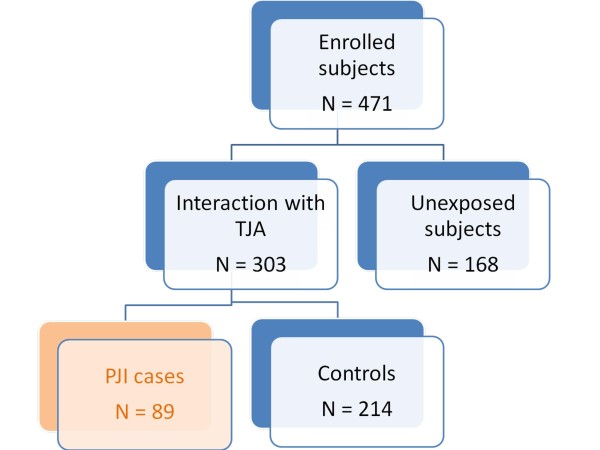
**Flowchart of the study design.** TJA – Total Joint Arthroplasty; PJI – Prosthetic Joint Infection.

In addition, 168 healthy people without TJA were recruited as a population control group [age, median (1st-3rd quartile): 28(24–34); males/females: 91/77]. All patients and controls were unrelated individuals of Czech Caucasian origin (Figure 1). The informed consent for the anonymous use of their DNA for the purposes of this study was obtained from all subjects. The study was performed with the approval of the local Ethics Committee.

### Phenotype definitions

Patients with PJI was diagnosed according to the previously published criteria [12]: 1) presence of sinus tract communicating with a joint and/or intra-articular pus; 2) coincidentally positive results of histological examination (five or more neutrophils per high power field) and culture of intraoperative samples; 3) if only intraoperative culture or histological results were positive, then high clinical suspicion of infection (either experienced as acute onset, fever, erythema, edema, and joint pain or persistent local pain, early prosthetic failure, history of wound healing disturbances, etc.) had to be present together with at least two of the following tests: erythrocyte sedimentation rate >30 mm/hr, C-reactive protein elevated more than 1.5 times above the laboratory reference value, positive 99m Technetium leukocyte scintigraphy (the latter criterion related only to the patients from the University Hospital Olomouc). The patients without PJI did not fulfil the above mentioned criteria for PJI.

### Candidate cytokine gene variants and genetic analysis

A crucial role of cytokines TNFalpha, IL-1beta and IL-6 in mediation and amplification of the immune response to bacterial pathogens, as described in the introduction, nominates the genes for these cytokines as highly plausible candidates for implication in the susceptibility to PJI. Accordingly, we selected six functional single nucleotide polymorphisms in the genes for TNFalpha (*TNF*-308, rs1800629; *TNF*-238, rs361525), IL-1beta (*IL1B*-511, rs16944; *IL1B* + 3962, rs1143634) and IL-6 (*IL6*-174, rs1800795; *IL6* nt565, rs1800797). Chosen SNPs became the most widely studied within particular cytokine genes for their possible association with diseases (http://www.ncbi.nlm.nih.gov/projects/SNP). The genotyping was performed in all subjects by means of the polymerase chain reaction with sequence-specific primers (PCR–SSP) using the original Heidelberg kit (Cytokine CTS – PCR-SSP Tray kit, product No. 124, University of Heidelberg, Heidelberg, Germany) according to the manufacturer’s instructions. Briefly, DNA was extracted from peripheral blood by the standard salting-out procedure [13] and obtained DNA was handled according to ethical rules. The PCR mix consisting of original “master” mix, Taq DNA polymerase (TopBio, Prague, Czech Republic) and template DNA was added to the lyophilised primers on the typing tray. The cycling conditions for PCR-SSP were as follows: 94°C - 2 min (initial denaturation), 10 cycles (94° - 15 sec, 65°C - 1 min) and further 20 cycles (94°C – 15 sec, 61°C – 50 sec, 72°C – 30 sec). PCR products were visualized on the agarose gel electrophoresis using an ethidium-bromide staining. The genotypes were determined based on the presence/absence of the allele specific and internal control bands. The nomenclature for the investigated SNPs was adopted from the Heidelberg kit manual. The typing kit was previously validated and used by us [14,15] and many others [16]. In particular, the genotype calling was tested in five of six investigated SNPs (except of *IL1B* + 3962) during the validation phase by “in-house” PCR-based methodologies with almost absolute concordance. The remaining SNPs from the Heidelberg kit (not selected for this study) were omitted from the analyses.

### Statistical analysis

Allelic and genotype frequencies and carriage rates were calculated for all investigated polymorphisms by direct counting [15]. The distribution of genotypes was tested for conformity with the Hardy–Weinberg equilibrium (HWE) using the chi-squared test [17]. The relationship between the genotypes and clinical/demographic variables was tested by chi-square test (gender, primary diagnosis/indication for TJA) or Mann–Whitney U test (age at index arthroplasty, body mass index). Statistical power of the study was calculated for each investigated SNP based on the allele frequencies in the healthy control group according to protocol described elsewhere [18].

Allelic, genotype and phenotype (carriage rates) frequencies were compared between the patients with PJI and both control groups using the standard chi-square test. The frequencies of the haplotypes for the pairs of *IL1B*, *TNF* and *IL6* SNPs were estimated by expectation–maximisation algorithm and the linkage disequilibrium (LD) was tested using the likelihood ratio test (software ARLEQUIN, version 3.000), [19]. In case of subanalysis within the group of patients with PJI (according to the type of PJI and the results of intraoperative culture) the Fischer exact test with two sided *p* values was used to analyze the differences between the groups. In all cases, a *p* value less that 0.05 was considered significant and corrected for the number of tested markers using the Bonferroni method.

## Results

To uncover any possible association between the functional polymorphisms of the cytokine genes and PJI, six selected SNPs (*IL1B*-511, *IL1B +* 3962, *TNF*-308, *TNF*-238, *IL6*–174, *IL6* nt565) were genotyped in the TJA patients with/without PJI and in the ethnically matched healthy controls. The distribution of genotypes for all investigated polymorphisms conformed to the HWE in both control groups (with non-significant chi-squared values); the distribution of *IL1B*-511 genotypes deviated from the HWE in the PJI group (p = 0.02). No significant relationship between genotypes of any of investigated SNPs and clinical/demographic variables (gender, body mass index, age at index arthroplasty, primary diagnosis/indication for TJA) was observed. Statistical power of the present study to detect a difference in minor allele frequency between the groups of TJA patients with / without PJI corresponding to odds ratio (OR) = 2 reached 98% for *IL1B*-511, 97% for *IL1B +* 3962, 94% for *TNF*-308, 61% for *TNF*-238, and 99% for both *IL6*–174 and *IL6* nt565 SNPs. The observed allelic and genotype frequencies and carriage rates for the investigated polymorphisms are summarized in Table 2.

**Table 2:** The genotype and allelic frequencies and carriage rates of six investigated single nucleotide polymorphisms (SNPs) of cytokine genes in 1) patients that developed prosthetic joint infection after total joint arthroplasty (TJA with PJI, N = 89), 2) patients without prosthetic infection (TJA without PJI, N = 214) and 3) the group of healthy control subjects (N = 168)

	**TJA with PJI**						**TJA without PJI**						**Healthy Controls (HC)**					
		**Genotype frequencies**		**Allelic frequencies**		**Carriage rates**		**Genotype frequencies**		**Allelic frequencies**		**Carriage rates**		**Genotype frequencies**		**Allelic frequencies**		**Carriage rates**
IL1B-511*	CC	0.31	C	0.61	C	0.91	CC	0.49	C	0.69	C	0.90	CC	0.45	C	0.68	C	0.91
	CT	0.60					CT	0.42					CT	0.46				
	TT	0.09	T	0.39	T	0.69	TT	0.10	T	0.31	T	0.51	TT	0.09	T	0.32	T	0.55
IL1B + 3962	CC	0.59	C	0.76	C	0.94	CC	0.57	C	0.75	C	0.92	CC	0.61	C	0.78	C	0.96
	CT	0.35					CT	0.35					CT	0.35				
	TT	0.06	T	0.24	T	0.41	TT	0.08	T	0.25	T	0.43	TT	0.04	T	0.22	T	0.39
TNF-308	GG	0.74	G	0.85	G	0.96	GG	0.72	G	0.85	G	0.97	GG	0.68	G	0.83	G	0.98
	GA	0.21					GA	0.25					GA	0.30				
	AA	0.04	A	0.15	A	0.26	AA	0.03	A	0.15	A	0.28	AA	0.02	A	0.17	A	0.32
TNF-238	GG	0.91	G	0.96	G	1.00	GG	0.93	G	0.96	G	1.00	GG	0.92	G	0.96	G	1.00
	GA	0.09					GA	0.07					GA	0.08				
	AA	0.00	A	0.04	A	0.09	AA	0.00	A	0.04	A	0.07	AA	0.00	A	0.04	A	0.08
IL6–174	GG	0.33	G	0.56	G	0.79	GG	0.36	G	0.58	G	0.79	GG	0.33	G	0.58	G	0.84
	GC	0.46					GC	0.43					GC	0.51				
	CC	0.21	C	0.44	C	0.67	CC	0.21	C	0.42	C	0.64	CC	0.16	C	0.42	C	0.67
IL6 nt565	GG	0.34	G	0.57	G	0.80	GG	0.38	G	0.59	G	0.81	GG	0.33	G	0.59	G	0.85
	GA	0.46					GA	0.43					GA	0.52				
	AA	0.20	A	0.43	A	0.66	AA	0.19	A	0.41	A	0.62	AA	0.15	A	0.41	A	0.67

The data are presented as relative values.

* *IL1B*-511*T carriage rate (Pearson’s Chi-square test, 1 degree of freedom, d.f.):

TJA with PJI *versus* TJA without PJI: p = 0.006, p_corr_ = 0.037; odds ratio = 2.06, 95%CI: 1.22-3.47.

TJA with PJI *versus* HC: p = 0.04, p_corr_ > 0.05; odds ratio = 1.76, 95%CI: 1.02-3.02.

* Distribution of *IL1B*-511 genotypes (Pearson’s Chi-square test, 2 d.f.):

TJA with PJI *versus* TJA without PJI: p = 0.014, p_corr_ > 0.05.

* Allelic frequency of *IL1B*-511*T (Pearson’s Chi-square test, 1 d.f.):

TJA with PJI *versus* TJA without PJI: p = 0.052.

**Figure 2:**
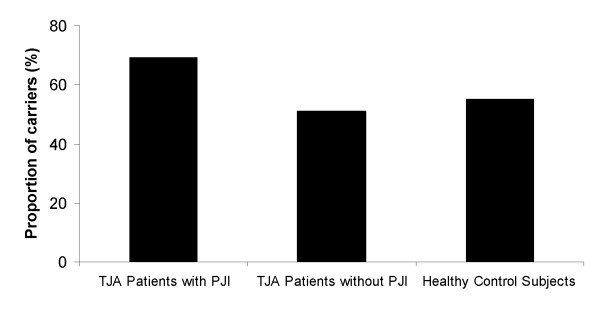
The proportion of the carriers of less common*IL1B*-511*T allele in the study groups. TJA – Total Joint Arthroplasty; PJI – Prosthetic Joint Infection. The p values and odds ratio for comparisons between the groups are provided in the Table 2.

Overall distribution of genotypes for *IL1B*-511 polymorphism differed nominally between TJA patients with PJI *versus* those without PJI (p = 0.014, p_corr_ > 0.05, 2 degrees of freedom), particularly due to the overrepresentation of *IL1B*-511 CT heterozygotes and reciprocal decrease of CC homozygotes among PJI patients. Importantly, the proportion of carriers of the less common *IL1B*-511*T allele was significantly higher in PJI patients (69%) than in those that did not develop PJI (51%, p = 0.006, p_corr_ = 0.037; odds ratio = 2.06, 95%CI: 1.22-3.47) or healthy controls (55%, p = 0.04, p_corr_ > 0.05; odds ratio = 1.76, 95%CI: 1.02-3.02), (Figure 2). Similarly, the allelic frequency of *IL1B*-511*T tended to be higher in the group of PJI patients by comparison to those with “aseptic” TJA (p = 0.052). The alleles and genotypes of the other five investigated cytokine gene polymorphisms were similarly distributed (p > 0.05) in all three groups (Table 2).The frequencies of two-locus haplotypes for each investigated cytokine gene estimated by expectation–maximisation algorithm are listed in Table 3. Consistent with the previous reports we detected strong LD between the alleles at two *IL6* SNP loci (IL6-174 / nt565, p < 0.00001 for all groups). The results from a single locus analysis in *IL1B* gene were reflected also in a haplotype level: The *IL1B −*511/ +3962 TC haplotype tended to be more frequent in PJI patients by comparison to those without infection after TJA. Nevertheless, overall haplotype distribution did not significantly differ between the PJI patients and those that did not develop PJI for any of investigated *IL1B*, *TNF* and *IL6* genes.

**Table 3:** Estimated frequencies of* IL1B, TNF *and* IL6 *two-locus (SNP) haplotypes in patients with prosthetic joint infection after total joint arthroplasty (TJA with PJI), those without infection (TJA without PJI) and Czech healthy controls. Data are given as proportions (relative numbers) of each possible haplotype

	**TJA with PJI**	**TJA without PJI**	**Healthy Controls**
***IL1B*haplotypes**			
**−511 / +3962**			
CC	0.398	0.476	0.479
CT	0.214	0.217	0.195
TC	0.367	0.272	0.304
TT	0.022	0.035	0.022
Overall LD *p* value	0.003	0.001	0.002
***TNF*haplotypes**			
**−308 / -238**			
AG	0.107	0.152	0.170
GA	0.045	0.040	0.042
GG	0.848	0.808	0.789
AA	0.000	0.000	0.000
Overall LD *p* value	0.607	0.289	0.017
***IL6*haplotypes**			
**−174 / nt565**			
CA	0.433	0.407	0.411
CG	0.011	0.016	0.006
GG	0.556	0.577	0.580
GA	0.000	0.000	0.003
Overall LD *p* value	<0.00001	<0.00001	<0.00001

LD – linkage disequilibrium.

For all comparisons of overall haplotype distribution between the groups: *p* > 0.05.

In order to reveal whether investigated polymorphisms of cytokine genes may be associated with PJI in particular anatomical localization we further compared their distribution separately in patients with total hip arthroplasty; this subanalysis could not be performed for knee prosthesis due to the very low number of control patients (N = 12). In compliance with the results in overall TJA groups (see Table 2), the patients with PJI of hip prosthesis (N = 39) carried *IL1B*-511*T allele in higher proportion (0.62) than those without PJI (N = 202, 0.50). Although this difference was almost the same as in comparison of overall TJA groups, it did not reach statistical significance due to the lower size of the subgroups.

In further subanalysis reflecting manifestation of infection after TJA, the PJI patients were subdivided according to the type of infection (early, delayed, haematogenous) and the results of intraoperative culture (Staphylococci, other pathogens, negative culture) as described in Table 1. Interestingly, the homozygotes for the less common *IL1B*-511*T allele (TT) were absent in the subgroup of PJI patients with culture positive for Staphylococci (0%) by comparison to those with other pathogens or a negative culture (23%, p_corr_ = 0.003). Reciprocally, the carriage of *IL1B*-511*C allele was significantly associated with the culture positive for Staphylococci among the PJI patients.

## Discussion

In the present study, we investigated the potential relationship between PJI and variability of the genes encoding for the key proinflammatory cytokines, namely IL-1beta, TNFalpha and IL-6. Of these, a particular *IL1B* gene promoter polymorphism was primarily nominated as the risk factor for PJI development. The arguments in favour of this interpretation are: i) adequate statistical power of the study; ii) biological relevance with IL-1β being a potent inducer of local inflammatory response; iii) the magnitude of statistical significance of the observed association [20]. In addition, we revealed an association between carrying of a particular IL-1 gene polymorphism and occurrence of a particular causative microorganism.

Few reports so far have described any association between the risk of PJI and genes related to the immune response. Malik et al. reported single centre data aimed at several targets. They investigated 71 septic cases and revealed the possible role of mannose-binding lectin (MBL) polymorphism in the pathogenesis of PJI [21]. These authors also associated PJI and polymorphism of the vitamin D receptor which might be in linkage disequilibrium with SNPs influencing on functionality of innate immunity [5]. On the other hand, they failed to reveal any association between septic failure of total hip arthroplasty and polymorphism in the genes encoding for IL-6 (as in our present study) and OPG/RANK/RANKL [5,22,23].

Cytokine IL-1beta exerts a wide range of inflammatory activities that are important in the context of the host defence against infection [8,24,25]. Although we have no direct explanatory evidence for an association of *IL1B* with the PJI found in our study, we can speculate that individuals carrying particular *IL1B* genotypes/ haplotypes differ substantially in their production of IL-1beta protein in response to infectious/ inflammatory stimuli [26,27]. In this regard, the effect of *IL1B* gene promoter polymorphisms and haplotypes to stimulated IL-1beta production *in vitro* has repeatedly been reported [28,29]. Besides we observed the association of *IL1B*-511*T allele with PJI in our study, the distribution of *IL1B* genotypes deviated from HWE in PJI group. We are aware that such finding may generally indicate problems in genotyping particularly in a “normal” population. However, we used the genotyping technique which was previously validated and successfully tested in parallel with an alternative procedure in our laboratory; furthermore, our both control groups (patients without PJI and healthy individuals) genotyped by the same technique were quite in HWE in regard to *IL1B* genotype distribution (*p* > 0.4 in both groups). From these reasons, a systematic bias introduced by a false genotyping is not probable and observed deviation from HWE may reflect real differences in the effects of particular *IL1B* genotypes on PJI susceptibility.

Concerning observed negative correlation between *IL1B*-511 TT genotype and the intraoperative culture positive for Staphylococci it is tempting to speculate on that staphylococci need less or even none host susceptibility to induce PJI compared to other pathogens. However, to confirm the above hypothesis, we would need to examine larger subgroups of patients. In fact, particular variants/ defects of immune response genes may confer susceptibility to infection caused by preferential spectrum of pathogens that is in extreme form evident in primary immunodeficiencies [30]. By analogy, pathogen(s) causative for PJI should always be taken into account in the studies searching for a genetic background of infectious complications after TJA. On the other hand, El-Helou et al. found no clinical association between TLR2 R753Q SNP and *Staphylococcus aureus* PJIs despite the *in vitro* demonstration that this SNP downregulates the TLR2-dependent immune and inflammatory response to *Staphylococcus aureus*[31].

The present study has limitations. First, the polymorphic variants of the immune-response genes are not the only independent variable modifying the anti-infection resistance of the patient. Several other factors have been nominated as influencing the risk for development of PJI. The research on the role of age, gender and other potential confoundings relevant to resistance/ vulnerability to PJI is still continuing [32-35]. For instance, aging should result in several structural and functional changes in the immune system ("immune senescence") which are generally associated with an increased risk for infection in elderly [36,37]. However, there is still lack of consensus regarding the age above which the immune system is more prone to PJI. In fact, several studies did not report the age being significant variable predicting development of PJI [2,38-40]. Regarding gender, some studies found a male gender to be more prone to PJI in comparison to female gender [32,41] while others did not confirm it considering male gender to be at least neutral variable regarding the risk for PJI [40,42]. The same has been reported for diabetes mellitus, rheumatoid arthritis and other potential confounders even that the effect of co-morbidities on the risk of PJI is widely accepted [34,40,43,44].

Additionally, due to its size this study may be underpowered for detection of small/moderate genetic effects on PJI susceptibility; this applies namely for the rare *TNF*-238 variant. Generally, genetic association studies present their own set of uncertainties and challenges that are deeply addressed in STROBE, STREGA, and recently GRISP statements [45,46], however we tried to address those in our study design & methodology, and also acknowledge that the observed association is of a preliminary character and specific for the Czech, Caucasian population.

### Perspectives for the future

There is no doubt that knowledge on a particular genetic trace increasing significantly the risk for PJI development could influence the outcomes of total joint arthroplasty. To achieve it there is necessary to increase the number of subjects/controls included in the study (multi-centre international collaborative projects) and to improve the data collected in such study (each host-, implant-surgery- and pathogen-related) with aim to reduce the potential influence of confounding on an observed association. Secondly, multiple potential targets should be analyzed together because it is likely that susceptibility to PJI is the result of polymorphisms from multiple genes rather than one single mutation. It can require testing simultaneously the set of at least thousand of genes that has been determined as involved in innate immune response. Finally, the research in the field of pathogenesis of PJI (infection) should be strongly supported in order to make genetic targets/mechanisms biologically plausible.

## Conclusion

In this study, we found an association between carriage of *IL1B*-511*T allele and increased risk for PJI development. We found no association between PJI and polymorphisms in the genes coding for TNFalpha and IL-6. Our data suggest that a functional allelic variant of the gene encoding for the cytokine IL-1beta may predispose patients after total joint arthroplasty to the development of PJI. This conclusion should, however, be considered preliminary until being independently replicated, preferably by multicentre efforts/multinational consortia.

## Competing interests

The authors declare that they have no competing interests’ that could have any bias on the outcomes of the study.

## Authors’ contribution

Conceptualization and design of the study was joint work of JG (clinical aspects of individual susceptibility to prosthetic joint infection) and MP (immunogenetic approach to prosthetic joint infection susceptibility). AS carried out the genotyping, performed statistical analysis (with help of FM and MP), and contributed to writing of the manuscript. FM contributed to the interpretation of genotyping, finalization of statistical analyses, and to writing of the manuscript. MS contributed to collection of clinical data; MP provided healthy population control group; contributed to statistical analysis and writing of the manuscript. JG recruited TJA patients into the study and contributed to collection of clinical details. Finalization and Editing of the manuscript was performed by JG, FM and MP. The author responsible for the integrity of the study is JG. All authors read and approved the final manuscript.

## Authors’ information

All authors are affiliated to the Faculty of Medicine and Dentistry, Palacky University, Czech Republic and, with exception to A.S. in parallel with the University Hospital Olomouc, Czech Republic. Anna Stahelova is a PhD student at the Laboratory of Immunogenomics & Imunoproteomics (LIGP); her tutor Assistant Professor Frantisek Mrazek, MD, PhD leads the DNA section of the Tissue Typing Laboratory and is the member of LIGP; Professor Martin Petrek, MD, PhD is the head of LIGP and the Director of the Tissue Typing Laboratory. Matej Smizansky, MD, PhD contributed to the study as a PhD student, his tutor Associate Professor Jiri Gallo, MD, PhD is the chief of Department of Orthopaedics.

## Grant support

IGA MZCR NS/10260-3.
